# Retention of Fœtus in a Sheep

**Published:** 1897-04

**Authors:** E. P. Niles

**Affiliations:** Virginia


### RETENTION OF FŒTUS IN A SHEEP.

By E. P. Niles, D.V.M.,
VIRGINIA.

I report herewith a case of retention of a fœtus in a sheep, not because similar cases are not on record, but because of the great length of time which the fœtus was retained.

*Subject:* A Dorset ewe, the property of the college farm. At one year of age it was thought that the ewe was pregnant, but date of breeding was not known. Some time later, however, the ewe showed less signs of being pregnant, and the manager, thinking that he had been mistaken about the ewe being in lamb, tried in vain to breed her for three years afterward, until February 24, 1897, the ewe being fat, she was slaughtered for mutton. Upon opening the abdominal cavity the butcher noticed that the uterus contained fluid, and was abnormally attached to the wall of the abdomen and a fold of the large intestine. What specially attracted his attention, however, was an unusually large nodule near one of the cornua of the uterus caused by the œsophagostoma columbianum. Being notified of the above condition I at once visited the slaughter-house. Upon opening the uterus a considerable quantity of a thin, fetid, apparently purulent fluid escaped. The os was firmly closed, and crowded well back against the os a number of small, flat bones (evidently the bones of the head), about the size of one's finger nail, were found.

The most remarkable thing in connection with this case is that fecundation took place three years ago, and that the ewe at no time showed any signs of parturition. Neither was the general health of the ewe at any time impaired to a sufficient extent to attract the attention of the attendants. At the time of slaughter she was exceedingly fat, and when hanging in the slaughter-house made as pretty mutton as one would wish to see. The carcass was condemned as unfit for human food.

